# A probabilistic model of pre-erythrocytic malaria vaccine combination in mice

**DOI:** 10.1371/journal.pone.0209028

**Published:** 2019-01-09

**Authors:** Erwan Atcheson, Karolis Bauza, Arturo Reyes-Sandoval

**Affiliations:** The Jenner Institute, University of Oxford, Oxford, United Kingdom; Rockefeller University, UNITED STATES

## Abstract

Malaria remains one the world’s most deadly infectious diseases, with almost half a million deaths and over 150 million clinical cases each year. An effective vaccine would contribute enormously to malaria control and will almost certainly be required for eventual eradication of the disease. However, the leading malaria vaccine candidate, RTS,S, shows only 30–50% efficacy under field conditions, making it less cost-effective than long-lasting insecticide treated bed nets. Other subunit malaria vaccine candidates, including TRAP-based vaccines, show no better protective efficacy. This has led to increased interest in combining subunit malaria vaccines as a means of enhancing protective efficacy. Mathematical models of the effect of combining such vaccines on protective efficacy can help inform optimal vaccine strategies and decision-making at all stages of the clinical process. So far, however, no such model has been developed for pre-clinical murine studies, the stage at which all candidate antigens and combinations begin evaluation. To address this gap, this paper develops a mathematical model of vaccine combination adapted to murine malaria studies. The model is based on simple probabilistic assumptions which put the model on a firmer theoretical footing than previous clinical models, which rather than deriving a relationship between immune responses and protective efficacy posit the relationship to be either exponential or Hill curves. Data from pre-clinical murine malaria studies are used to derive values for unknowns in the model which in turn allows simulations of vaccine combination efficacy and suggests optimal strategies to pursue. Finally, the ability of the model to shed light on fundamental biological variables of murine malaria such as the blood stage growth rate and sporozoite infectivity is explored.

## Introduction

Despite substantial reduction in the morbidity and mortality due to malaria over the past fifteen years [1], this parasitic disease remains one of the leading global causes of mortality caused by infection, with almost half a million deaths and 148–304 million clinical cases every year [2]. An effective vaccine against malaria is more urgently needed than ever. The most advanced malaria vaccine, RTS,S, (based on the CSP antigen) provides only modest efficacy of 30%-50% under field conditions [3]. Other subunit vaccines show similar low efficacy (for instance, based on the TRAP antigen [4–6]), which has led to interest in combining malaria subunit vaccines to improving protective efficacy. Such combinations have shown promise in pre-clinical murine studies [7–9].

Mathematical models of the effect of combining such vaccines on protective efficacy can help inform optimal vaccine strategies and decision-making [10–14]. So far, however, no model has been developed for pre-clinical murine studies, the stage at which candidate antigens begin evaluation. To facilitate the translation of findings from pre-clinical to clinical trials this paper develops a novel mathematical model of vaccine combination adapted to murine malaria studies, expressing the probability of sterile protection as a function of antibody and cytotoxic T-cell levels. Thus a framework is established within which combination vaccine experiments can be evaluated and interpreted. The model also furnishes a theoretical basis upon which combination vaccines might be expected to enhance efficacy.

To place this pre-erythrocytic model of vaccine efficacy in context, the life-cycle of malaria is now briefly outlined. On taking a blood meal, female Anopheline mosquitoes infected with malaria deposit sporozoites from their salivary glands into the host dermis, from which the sporozoites migrate to capillaries to enter the blood [15,16]. Very soon [17–19] after entry, sporozoites arrest in the liver [20,21] and invade hepatocytes. The parasite replicates over the course of two days in mice or up to eight days in humans [22,23], forming 1000 ([24,25], mice) or tens of thousands of merozoites ([23], humans) which, upon rupture of the hepatocyte, enter the blood and rapidly invade red blood cells, initiating cycles of replication inside red blood cells, egress, and reinvasion of red blood cells [23,26–29]. Depending on *Plasmodium* species, the sexual forms of malaria are formed immediately after initiation of the blood stage of infection or a few weeks later [23,30]. These are then taken up by a mosquito where they develop to complete the life-cycle [23,31]. Vaccines have been developed against almost all stages of the life-cycle of malaria, with pre-erythrocytic vaccines targeting the sporozoite as it moves from the dermis to the liver, or targeting infected hepatocytes themselves. Vaccines such as RTS,S and R21 function primarily by generating CSP-specific neutralising antibodies against the sporozoite though T-cell mediated immunity is also thought to play a role [32]. Vaccines such as the viral-vectored ME-TRAP function primarily by generating high numbers of cytotoxic T-cells which can recognise and eliminate infected hepatocytes [4,5,33,34]. Whole sporozoite vaccines have more complex modes of protection involving cellular and humoral immunity against a range of pre-erythrocytic malarial antigens [35]. The model here-in developed considers vaccines acting in simple single-mechanism fashions, but the principles of multi-mechanism protection described will apply to all pre-erythrocytic vaccine modes of action.

Three models of human malaria vaccine combination have been developed for clinical or field conditions, by Saul *et al*., [10,11], White *et al*., [12,13], and Walker *et al*., [14]. White *et al*. [12,13] follow Saul *et al*. [10,11] in developing a model based on the assumption that the dose-response relationship between adaptive immune responses and the probability of a sporozoite surviving those can be modelled using a Hill function. White *et al*. consider as an alternative that the relationship can be modelled using an exponential dose-response relationship. Here we’ll consider the exposition in [12] as it lucidly explicates both models.

For the Hill function and exponential models, the parameters of interest are *x*_*ij*_, the antibody titre to a specific antigen *j* in a given individual *i*; *r*_*ij*_, the probability that a sporozoite survives the immune response *x*_*ij*_; and *β*_*j*_, the antibody titre which reduces the probability of sporozoite infection by half. If we assume that a Hill-function captures the relationship between *x*_*ij*_ and *r*_*ij*_ then the model is

rij=11+(xijβj)α

where α is the shape parameter. If the dose-response relationship is assumed to be exponential, on the other hand, then the relationship is

$$r_{ij}=e^{-x_{ij}\log\left(2\right)/\beta_{j}}$$

From this core, White *et al*. and Saul *et al*. expand the two models to include the variation in numbers of sporozoites inoculated per bite and variation in antibody responses and biting rates.

Walker *et al*. [14] present a third model, explicitly designed to capture the effects on protective efficacy of CSP-specific antibodies and TRAP-specific cytotoxic T-cells. Here four equations are assumed to capture the dynamic effects of adaptive immunity on infection:

$$\frac{\text{d}H}{\text{d}t}=-\alpha_{2}H$$

$$\frac{\text{d}M}{\text{d}t}=\gamma M$$

$$H_{0}=\left(1-\alpha_{1}\right)fS_{0}$$

$$M_{T}=rH_{T}$$

where *H* is the number of infected hepatocytes at time *t*; α_2_ the vaccine-induced rate of removal of infected hepatocytes; *M* the number of merozoites at time *t*; *γ* the blood-stage growth rate; *H*_*0*_ the initial number of infected hepatocytes at the beginning of the liver-stage of the life-cycle; α_1_ the vaccine-induced modifier of sporozoite invasion probability; *f* the proportion of sporozoites that successfully invade hepatocytes; *S*_*0*_ the number of sporozoites inoculated; *M*_*T*_ the number of merozoites seeding the blood stage of infection; and *r* the number of successful merozoites released per infected hepatocyte *H*_*T*_ after the liver-stage incubation period *T*. Walker *et al*. also expand the basic model to adapt it to CHMI. As with the other models, that of Walker *et al*. is found to fit real CHMI data well, and predicts that a combination of CSP and TRAP vaccines will enhance protective efficacy.

The model developed in this paper closely follows the work of White *et al*., Saul *et al*. and Walker *et al*., but adapted to murine systems and deriving the equations from more fundamental biological assumptions, including a probabilistic interpretation of sporozoite infectivity owed to [36]. One difference is that the present model simplifies the sporozoite-inocula component of the models, to make it more usable for murine studies while at the same time taking into account the wide variation in sporozoite infectivity between murine challenge experiments; it is equivalent to an exponential dose-response model.

The second major difference between the model presented in this paper and the models of White *et al*., Saul *et al*. and Walker *et al*. is an attempt to derive a relationship between the level of adaptive immune components (antibodies and cytotoxic T-cells) and sporozoite survival from very simple probabilistic assumptions and by so doing place the modelling the combination of vaccines on a firmer theoretical footing.

In this paper, a second metric of vaccine efficacy besides sterile protection, time taken to reach 1% blood stage parasitaemia, is also modelled as a function of antibody and cytotoxic T-cell quantities. Pre-erythrocytic vaccines, if they have partial efficacy, reduce the number of merozoites seeding the blood stage of infection by reducing the number of infected hepatocytes [37]. This can result in a delay in time to reach 1% blood-stage parasitaemia [34]. Given the current practice of benchmarking human malaria vaccines against existing vaccines that frequently confer complete protection, the utility of extending the model to encompass time to 1% parasitaemia as a metric of vaccine efficacy could be questioned. However, when conducting pre-clinical work to investigate the combination of malaria vaccines, time to reach 1% parasitaemia provides a more sensitive measure of whether combination actually enhances protective efficacy, and requires fewer mice. This can be seen in a recent paper where two malaria vaccines, Rv21 and TRAP, were titrated to suboptimal doses in order that an enhancement of their combined protective efficacy could be seen [9].

To achieve an expression containing this metric of pre-erythrocytic vaccine efficacy, a simple model of blood-stage malaria growth is used. There are many available models, ranging from very simple exponential growth models [14,38] to those modified to take account of sequestration or periodicity [39,40], erythrocyte kinetics [41], and others [37,40–47]. To develop the model presented in this paper, the most simple exponential growth model was chosen. This was motivated by considerations of tractability and by the fact that the metric of challenge outcome used in this paper, time-to-1%, is based on the simple exponential growth model. Above all the use of the simpler exponential growth model is justified by the finding in [38] that it is as accurate in predicting parasitaemia levels in CHMI studies as more complex models.

In short, a theoretical basis for the enhancement of efficacy obtained with vaccine combination regimes is derived from simple biological assumptions. Variables such as the blood-stage growth rate in the murine malaria model are estimated within in the context of the model, and used to generate simulations informing optimal strategies for the combination of pre-erythrocytic malaria vaccines.

## Results

### Derivation of a probabilistic equation modelling pre-erythrocytic malaria vaccine efficacy

Here we derive an equation representing the protective effects of antibody and cytotoxic T-cell (CTL)-eliciting vaccines from minimal probabilistic assumptions. For ease of exposition, equations are numbered and the symbolic representations highlighted in bold immediately following or preceding their definition. Also see Table 1 for a complete list of definitions of symbols used in this section.

**Table 1:** List of variables.

*p*_*S*_	probability of sterile protection
*k*	natural infectivity of sporozoites
*p*_*b*_	probability that a unit of antibody prevents successful infection
*b*	quantity of antibody
*p*_*C*_	probability that a unit of CTL prevents successful infection
*C*	quantity of CTL
*Z*	number of sporozoites in challenge

The assumptions are:

i) a unit of monoclonal antibody or CTL has a fixed probability (***p***_***b***_ or ***p***_***c***_ respectively) of preventing successful infection by a given sporozoite. "Successful infection" denotes the event where a given sporozoite invades a hepatocyte and fully develops to initiate blood-stage infection.
ii) the probabilities *p*_*b*_ and *p*_*c*_ and the probability ***p***_***Z*,**_ that a given sporozoite successfully infects, are independent.

The aim is to derive an expression of the probability of sterile protection ***p***_***S***_ as a function of the number of units of antibody ***b***, CTL ***C***, number of sporozoites ***Z*** and unit probabilities of preventing successful invasion *p*_*b*_ and *p*_*c*_.

To achieve sterile protection, each sporozoite must fail to initiate blood stage infection. The probability that a given sporozoite fails to infect is (1–*p*_*Z*_), and hence the probability *p*_*S*_ that all sporozoites fail to infect is

$$p_{S}={\left(1-p_{Z}\right)}^{Z}$$ (1)

The probability that a given sporozoite infects is a function of its natural infectivity ***k*** and the probability that the antibodies and CTL in the system all fail to prevent infection. Here I interpret natural infectivity probabilistically: in the absence of antibodies or CTL, a given sporozoite has a probability *k* of successful infection. In the absence of antibodies or CTL, *p*_*Z*_ *= k*.

The probability *p*_*Z*_ that a given sporozoite will successfully infect in the presence of antibodies and CTL is the product of the probability that each unit of antibody and each unit of CTL fails to prevent infection, and the natural infectivity. The probability that every unit *b* of antibody fails to prevent a given sporozoite infecting is (1–*p*_*b*_)^*b*^ and likewise the probability that every unit *C* of CTL fails to prevent a given sporozoite infecting is (1–*p*_*c*_)^*C*^. Hence the probability that all units of antibody and CTL fail, and that a given sporozoite infects, is

$$p_{Z}=k*{\left(1-p_{b}\right)}^{b}*{\left(1-p_{C}\right)}^{C}$$ (2)

From **(1)** and **(2)** we get

$$p_{S}={\left(1-\left(k*{\left(1-p_{b}\right)}^{b}*{\left(1-p_{C}\right)}^{C}\right)\right)}^{Z}$$ (3)

### Time to 1% blood-stage parasitaemia as a function of antibody and CTL

As well as conferring sterile protection, effective pre-erythrocytic vaccines cause a delay in time to patency by decreasing the number of infected hepatocytes initiating the blood stage of infection. Here we derive equations expressing the time taken to reach a given level of blood-stage infection as a function of the quantity of antibodies and CTL. To this end we have assumed a simple model of growth: at time ***t*** from the beginning of blood-stage infection, the number of infected red blood cells (**iRBC**) is given by

$$iRBC=M*\left(g^{t}\right)$$ (4)

where ***M*** is the number of infected red blood cells seeding the blood stage infection at time *t* = 0 and ***g*** is the growth rate, which is the daily blood-stage parasite replication rate; because the cycle of replication in mice is approximately one day, this is also the average number of infected red blood cells generated from each infected red blood cell. See Table 2 for a full list of variables used in this section.

**Table 2:** List of variables.

*M*	Number of iRBC initiating blood-stage infection
*B*	Number of RBC in mouse
*R*	Number of iRBC produced per infected hepatocyte
*L*	Number of successfully infected hepatocytes
*L*_*V*_	Number of successfully infected hepatocytes in vaccinated mouse
*L*_*N*_	Number of successfully infected hepatocytes in naive mouse
*T*	Time to 1% blood-stage parasitaemia
*T*_*V*_	Time to 1% blood-stage parasitaemia in vaccinated mice
*T*_*N*_	Time to 1% blood-stage parasitaemia in naive mice
*g*	growth rate of blood stage parasite

A common metric by which challenge outcome in murine malaria studies is assessed is ***T***, time taken post-infection to reach 1% blood-stage parasitaemia; hereafter truncated to “time-to-1%”. Since *t* in **(4)** refers to time after the beginning of blood-stage infection, and *T* refers to time taken to reach 1% iRBC after sporozoites are injected into a mouse, to substitute *T* into **(4)** we need to take into account the time for pre-erythrocytic development, ***t***_***1***_. If the number of red blood cells (RBC) in the system is ***B***, then iRBC = 1% = *B*/100 when *t = T–t*_*1*_. Then from **(4)** we get:

$$M=\frac{B}{100*g^{T-t_{1}}}$$ (5)

The number of iRBC initiating blood-stage infection, *M*, is the product of the number of infected hepatocytes contributing to the blood stage, ***L***, and the average number of iRBC each infected hepatocyte contributes, ***R***: *M* = L*R. Therefore

$$L=\frac{B}{100*R*g^{T-t_{1}}}$$ (6)

The number of successfully infected hepatocytes, *L*, is in turn the product of the probability that a given sporozoite infects, *p*_*Z*_, and the number of sporozoites injected, *Z*: *L* = *p*_Z_*Z. If in a given experiment ***L***_***N***_ represents the average number of infected hepatocytes in naive mice and *T* = ***T***_***N***_, and ***L***_***V***_ represents the number of infected hepatocytes in the vaccinated mouse with *T =* ***T***_***V***_, then from **(2)** we obtain

$$L_{N}=Z*k$$ (7)

$$L_{V}=Z*k*{\left(1-p_{b}\right)}^{b}*{\left(1-p_{C}\right)}^{C}$$ (8)

and from **(6)** we obtain

$$L_{N}=\frac{B}{100*R*g^{Tn-t_{1}}}$$ (9)

$$L_{V}=\frac{B}{100*R*g^{Tv-t_{1}}}$$ (10)

Therefore

LVLN=(1−pb)b*(1−pc)C=(B100*R*gTv−t1)(B100*R*gTn−t1) (11)

If we assume that *B* and *R* are identical in vaccinated and unvaccinated mice, then

$${\left(1-p_{b}\right)}^{b}*{\left(1-p_{c}\right)}^{C}=g^{Tn-Tv}$$ (12)

By taking the logarithm of **(12)** we obtain *T*_*V*_ as a function of quantity of antibody and CTL in the system:

$$T_{V}=-\frac{\log\left(1-p_{b}\right)}{\log\;g}*b-\frac{\log\left(1-p_{c}\right)}{\log\;g}*C+T_{N}$$ (13)

If vaccine effects are mediated exclusively by antibody or CTL, that is if *b* or *C* = 0, we obtain the following linear equations:

$$T_{V}=-\frac{\log\left(1-p_{b}\right)}{\log\;g}*b+T_{N}$$ (14)

$$T_{V}=-\frac{\log\left(1-p_{c}\right)}{\log\;g}*C+T_{N}$$ (15)

### Experimental estimates of *g*, *p*_*b*_ and *p*_*c*_

#### Estimating g

The growth rate *g* of blood-stage parasitaemia may not be constant over the entire course of infection, or between the initiation of blood-stage infection and 1% blood-stage parasitaemia. However, for the purposes of the model described in this paper, it is important only that the effective growth rate *g* averaged between the initiation of blood-stage infection and *t* = *T* is invariant between mice, vaccinated or otherwise.

**Fig 1:**
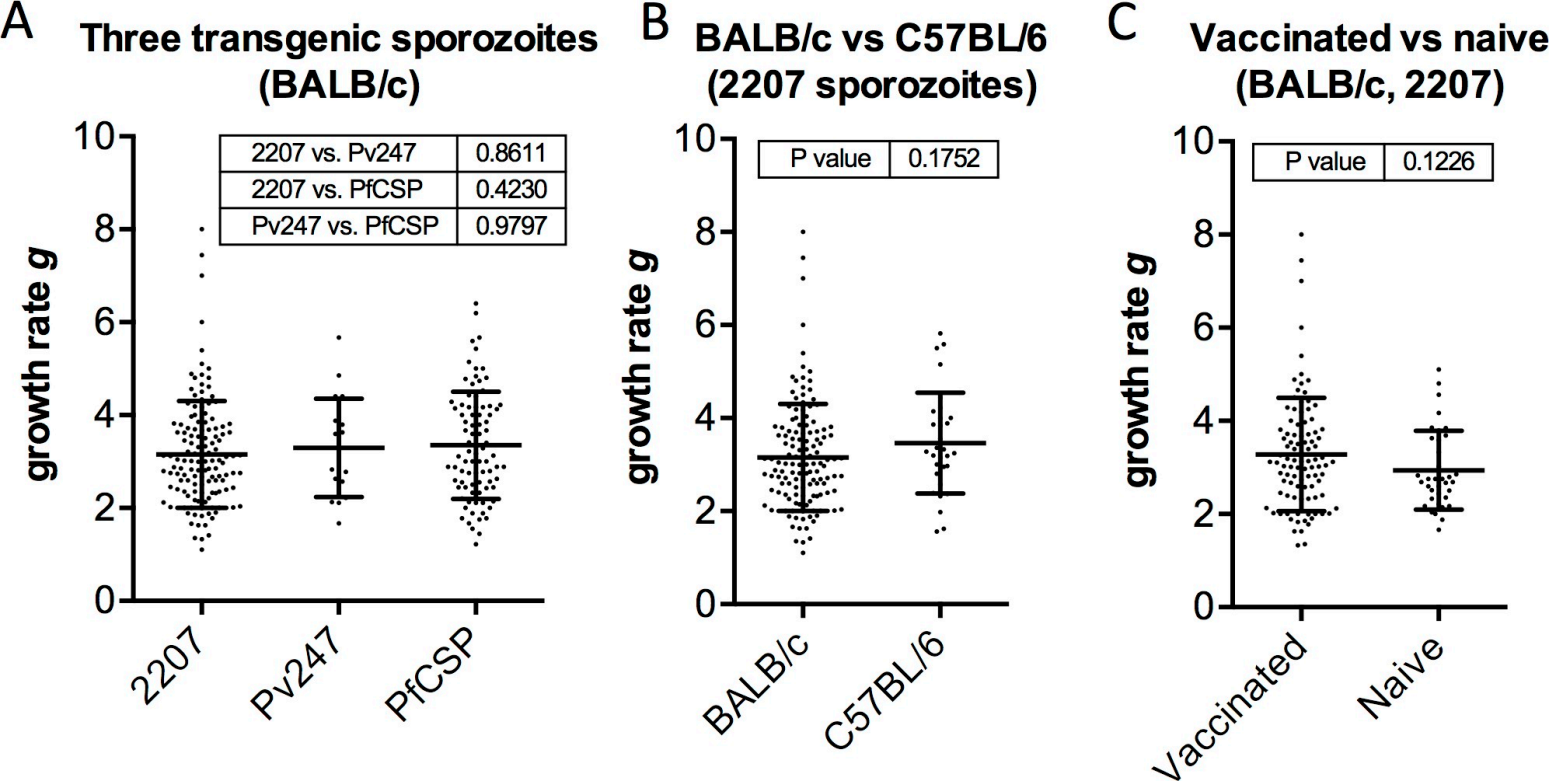
Estimates of blood-stage growth rate g from thin blood smears taken 24 hours apart. Thin blood smears (n = 273) were taken one day either side of 1% blood-stage parasitaemia for (A) three different transgenic *P*. *berghei*; (B) two strains of mouse using PvCSP-210/PvTRAP transgenic *P*. *berghei* (“2207” parasites); and (C) vaccinated and unvaccinated BALB/c mice using PvCSP-210/PvTRAP transgenic *P*. *berghei*. Vaccinated mice were administered CSP- or TRAP-based subunit vaccines. Growth rate *g* was calculated by dividing parasitaemia on the day after reaching 1% infected red blood cells by that of the previous day. Shown in tables in each graph are p-values for comparison of groups by (A) ANOVA with Tukey’s multiple comparisons test and (B, C) t-test. No significant differences between groups were found. The data together give a value for *g* = 3.26 ± 0.07 (SEM).

Some sporozoite-based vaccines do generate immunity against blood-stage antigens [48]. However, most pre-erythrocytic subunit vaccines are thought not to. We have vaccinated many mice with CSP- and TRAP-based subunit vaccines in the course of our studies. That these pre-erythrocytic vaccines do not affect blood-stage growth rate is suggested by the blood-stage growth rate as inferred from parasitaemia counts of thin blood smears taken 24 hours apart, either side of time-to-1% (Fig 1). This demonstrates that the average growth rate, defined as the ratio of % parasitaemia of one day to the previous, is not significantly different when using different transgenic *P*. *berghei* sporozoites (Fig 1A), between BALB/c and C57BL/6 mice (Fig 1B), or, crucially, between vaccinated and naive BALB/c mice (Fig 1C). These data together give a value of *g* = 3.26 ± 0.07.

An estimate of *g* from blood smears around time *T* will not necessarily give us an accurate value as *g* may not be constant between the initiation of blood-stage infection and *t* = *T*. An alternative approach is to seed the blood-stage infection with a dilution series of iRBC (*M*) and curve fit a modified form of Eq **(5)** to a plot of *M* against *T+0*.*5*. (*t*_*1*_ = 0.5 d as the population of iRBC from donor mice will be at various stages of the 24 h replicative cycle; if the distribution is balanced, the mean will be 0.5 d).

$$M=\frac{B}{100*g^{T+0.5}}$$ (16)

**Fig 2:**
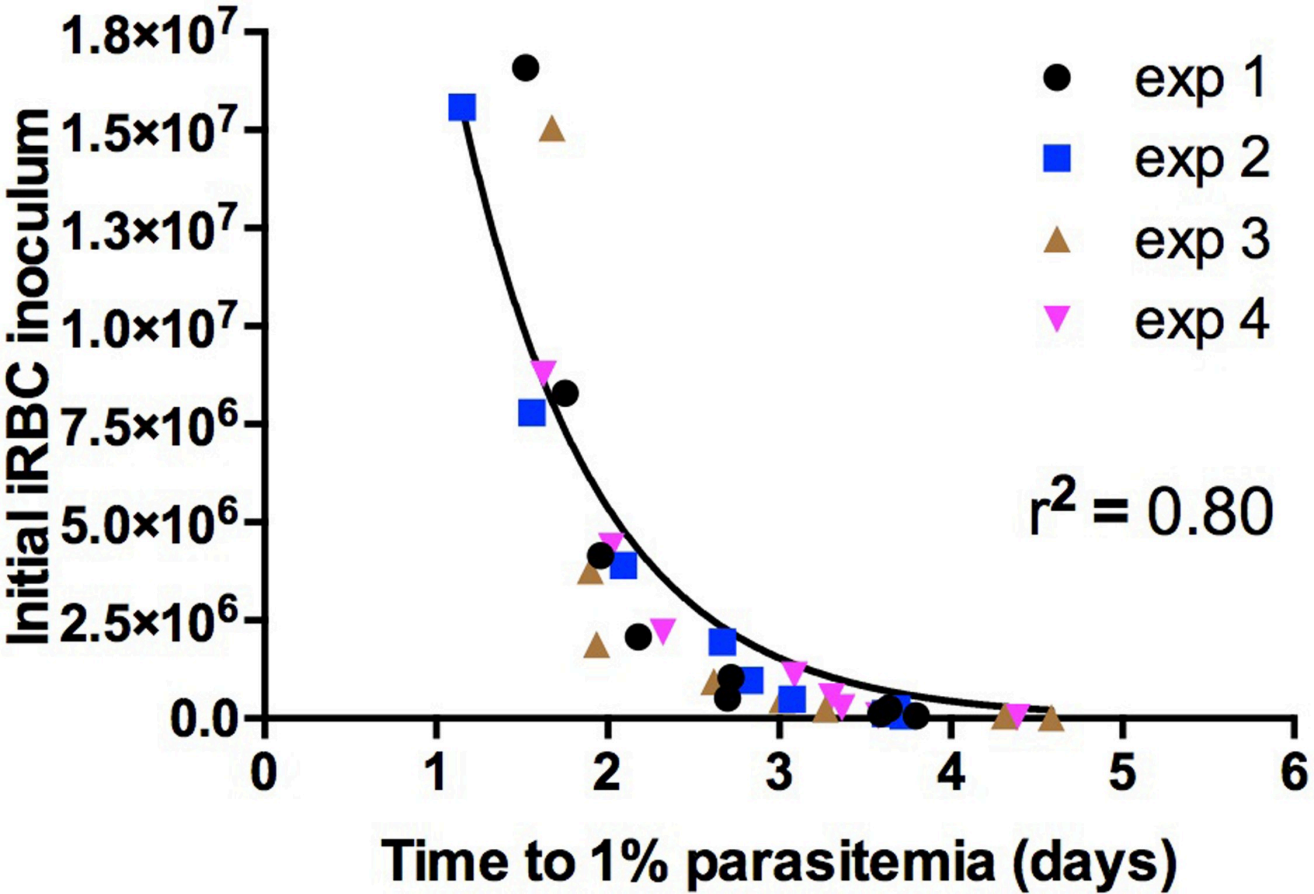
Seeding blood-stage parasitaemia with infected red blood cells (iRBC) to obtain an estimate of blood-stage growth rate by non-linear curve fitting. Dilution series of PvCSP-210/PvTRAP transgenic *P*. *berghei* iRBC were intravenously injected into 6–8 week old BALB/c mice (n = 34) and time to 1% blood stage parasitaemia inferred by linear regression from daily thin blood smears. Curve-fitting to the equation $M=\frac{B}{100*g^{T+0.5}}$ was performed using Prism to obtain a value for the growth rate *g* where *M* is the number of iRBC injected, *T* time-to-1% in the challenged mouse, and *B* the average number of RBC in a 22 g BALB/c mouse. A value of *g* = 3.50 ± 0.12 (SEM) was obtained.

Fig 2 shows the results of four independent experiments. Fitting the curves with the question of whether *g* was shared between each data set or not returned the conclusion that *g* was shared with a p-value of 0.57. The subsequent global value of *g* is 3.50 ± 0.12. Curve-fitting gives much lower variance in *g* than the blood-smear method (Fig 1), suggesting that the high variance found using that approach is due to experimental error.

Curve-fitting to Eq **(16)**, though producing a relatively precise value of *g*, may still not be accurate. The merozoites from the mice used as the source of the iRBC in the above experiments were obtained when blood-stage parasitaemia was 1% or higher; conceivably merozoites at this stage could have phenotypic differences from those emerging from the liver in a natural infection which could affect *g*. A method which perfectly recapitulates natural infection to give an accurate value of *g* is curve fitting to the following equation derived from **(6)** and **(7)**:

$$Z=\frac{B}{100*R*k*g^{T-t_{1}}}$$ (17)

Although *R* and *k* are unknowns and *k* at least will vary between experiments, *g* can be determined independently of *R* and *k* as can be more easily seen in a linear version of **(6)** where a plot of *T* against log(*Z*) will have slope -1/log(*g*):

T=−1logg*logZ+(t1+logB100*R*klogg) (18)

As well as **(6)** there is a second equation linking *Z* to *T* to which non-linear curve fitting could be applied. There will be a time *T*_*max*_ representing the longest possible time to 1% parasitaemia, occurring when just one infected hepatocyte seeds the blood-stage infection. The ratio of *L*_*N*_/(*L = 1*) gives

$$Z=\frac{g^{Tmax-Tn}}{k}$$ (19)

If estimating *g* is the objective it makes no difference which equation is used for curve-fitting.

**Fig 3:**
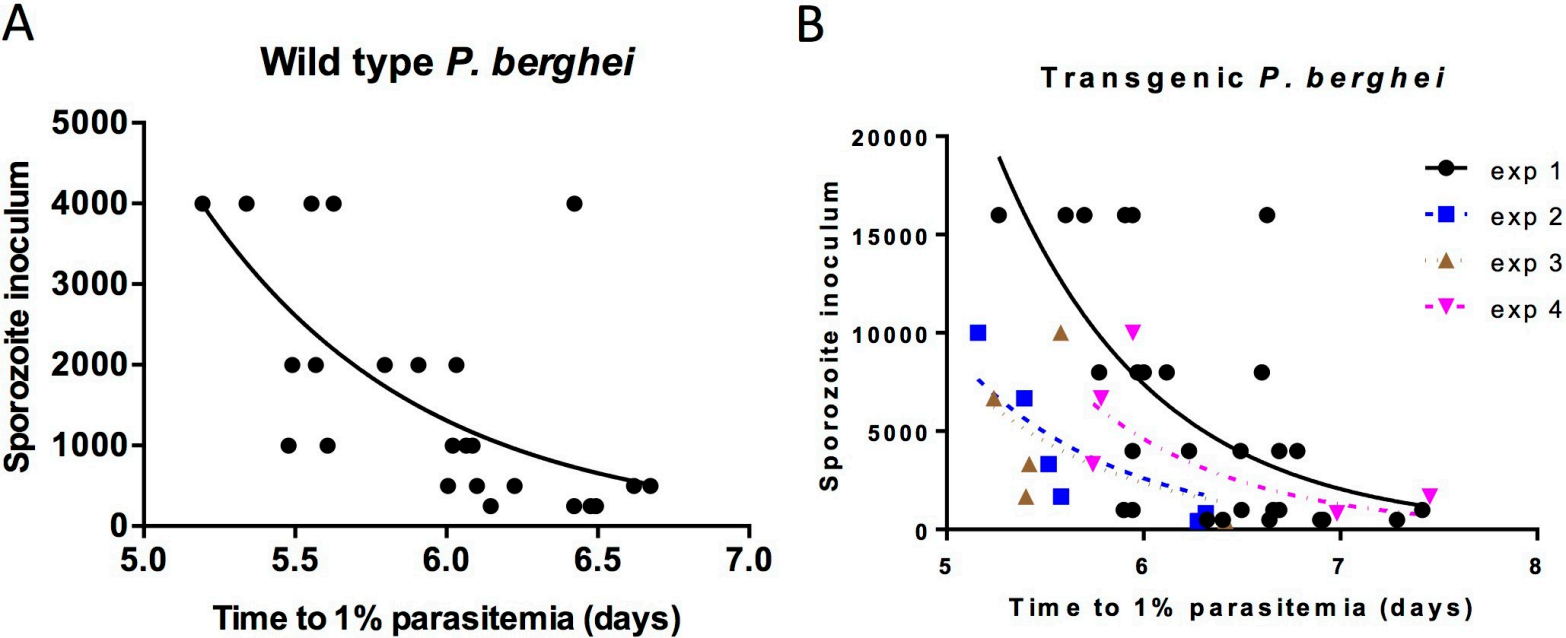
Varying *P*. *berghei* sporozoite inoculation to obtain an estimate of blood-stage growth rate by non-linear curve fitting. Dilution series of wild-type (A) or PvCSP-210/PvTRAP transgenic (B) *P*. *berghei* were intravenously injected into 6–8 week old BALB/c mice and time to 1% blood stage parasitaemia was inferred by linear regression from daily thin blood smears. Curve-fitting to the equation $Z=\frac{g^{Tmax-Tn}}{k}$ was performed using Prism to obtain a value for the growth rate *g* where *Z* is the number of sporozoites injected, *T*_*max*_ maximum time-to-1%, *T*_*N*_ time-to-1% in a challenged mouse, and *k* the infectivity of sporozoites. A value of *g* = 4.0 ± 1.5 was obtained from (A, n = 24) and *g* = 3.6 ± 0.9 from (B, n = 46). Best fit values given ± SEM.

Challenging mice with a dilution series of sporozoites and measuring the subsequent values of *T* thus permits a potentially more accurate method of estimating *g*. Fig 3 shows two experiments to which this method is applied, one using wild-type *Plasmodium berghei*, and the other using a double transgenic Pv210/PvTRAP parasite (2207) used in Fig 2.

Curve-fitting was successful using both wild-type and transgenic *P*. *berghei* and gave values of *g* = 4.0 ± 1.5 and *g* = 3.6 ± 0.9. Instructed to test whether *g* was shared between all five data sets (one wild type *P*. *berghei* and four from 2207 transgenic *P*. *berghei*) Prism best fits the data on the assumption that *g* is shared (p = 0.86). The combined value for *g* is 3.6 ± 0.73.

**Fig 4:**
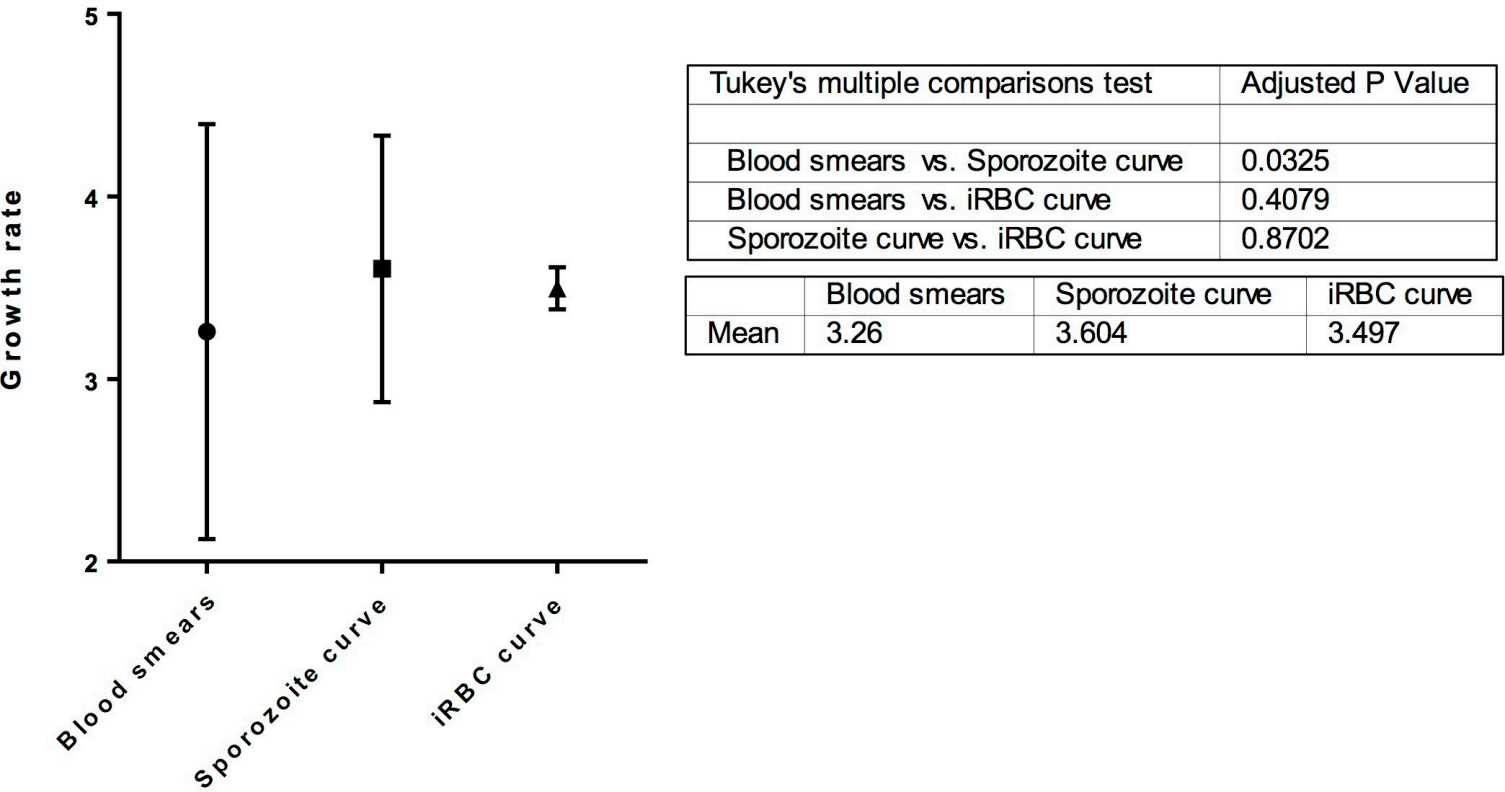
Comparison of methods of calculating growth rate *g*. Values for *g* were obtained using thin blood smears (n = 273), curve fitting to a dilution series of intravenously injected sporozoites (n = 70), and to a dilution series of intravenously injected infected red blood cells (iRBC) (n = 34). Comparison of methods was performed using ANOVA with Tukey’s multiple comparisons test. P-values are shown in the table, together with mean values for *g* by each method.

A comparison of all three methods of calculating *g*, using thin blood smears, or varying iRBC or sporozoite inocula, is shown in Fig 4. The values for *g* obtained from varying sporozoite and iRBC inocula are in close agreement and not significantly different from each other. The value for *g* from thin blood smears is significantly lower than that from varying sporozoite inocula, suggesting that by the time 1% parasitaemia is reached, growth may be slowing slightly, possibly as reticulocytes, the preferred blood cell type for invading merozoites, become scarcer. Since the *g* values obtained from sporozoite and iRBC curves are in close agreement with each other, and that of the iRBC curve is relatively precise, the value from the latter curve-fitting of *g* = 3.5 ± 0.1 is used for the remainder of this paper.

#### Estimating pb

*p*_*b*_ can be estimated by varying the quantity of antibody in a system and challenging with a fixed dose of sporozoites. If monoclonal or polyclonal antibody are injected we can be certain all vaccine effects are due to antibody alone; that is, *C* = 0 and from **(3)**:

$$p_{S}={\left(1-\left(k*{\left(1-p_{b}\right)}^{b}\right)\right)}^{Z}$$ (20)

And Eq **(14)** shown earlier. These two equations allow independent methods for estimating *p*_*b*_. The first method requires injecting groups of mice with varying quantities of antibody and curve-fitting **(20)** to a plot of percent protection against quantity of antibody *b* injected. The second method exploits time-to-1% (*T*) data from the same experiment: a plot of *T*_*V*_ against *b* will give a straight line of slope (log(1–*p*_*b*_))/(-log(*g*)) as shown in **(14)**. By using the value of *g* estimated in the section above, we can obtain another estimate of *p*_*b*_.

**Fig 5:**
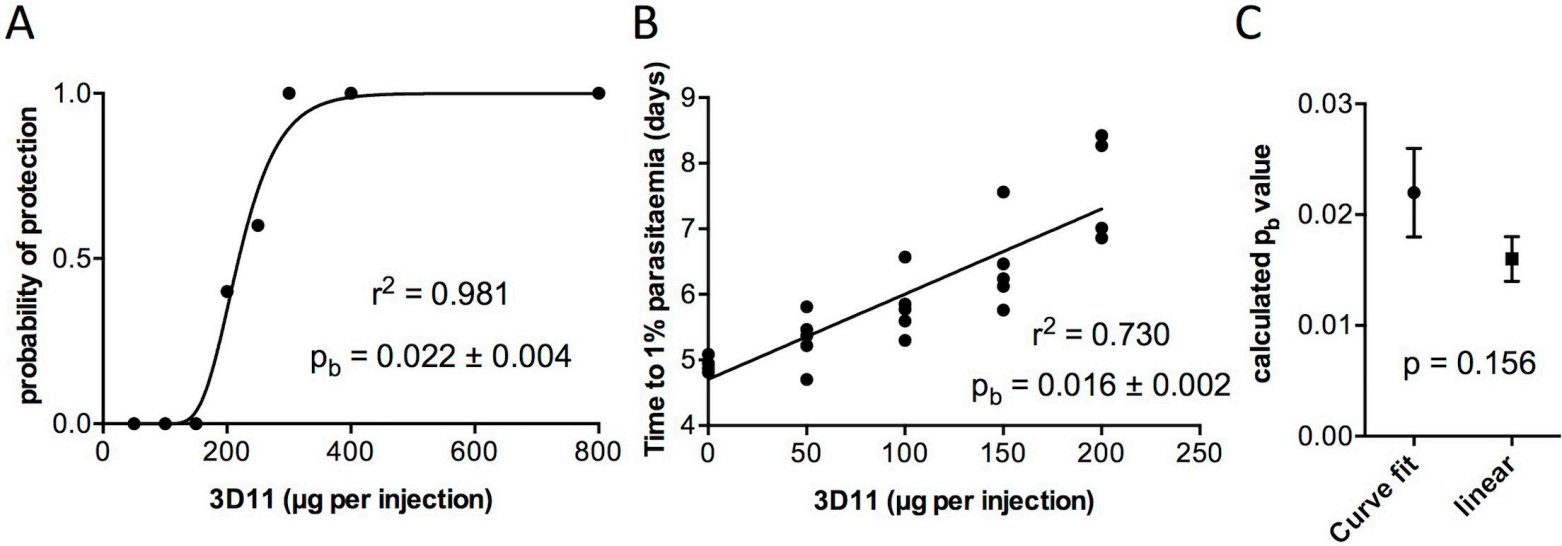
Varying monoclonal antibody 3D11 dose to obtain estimates of probability of protection parameter *p*_*b*_. Mice were injected on each of three consecutive days with varying quantities of the *P*. *berghei* sporozoite-neutralising monoclonal antibody 3D11 prior to intravenous challenge with 2000 wild-type *P*. *berghei* sporozoites (n = 5–6 per group). (A) A value for the probability of protection parameter *p*_*b*_ was obtained by non-linear curve fitting to $p_{S}={\left(1-\left(k*{\left(1-p_{b}\right)}^{b}\right)\right)}^{Z}$, where *p*_*S*_ is the probability of sterile protection, *b* the quantity of 3D11 monoclonal antibody injected, and *Z* the number of sporozoites used to challenge the mice. (B) An alternative value for *p*_*b*_ was obtained by linear regression of a plot of [3D11] versus time to reach 1% blood-stage parasitaemia (obtained by linear regression using daily thin blood smears); *p*_*b*_ was calculated from the slope using the equation $T_{V}=-\frac{\log\left(1-p_{b}\right)}{\log\;g}*b+T_{N}$. (C) Comparison of *p*_*b*_ values obtained from (A) and (B), using t-test. Values shown ± SEM.

The following estimate of *p*_*b*_ is derived from data using the *P*. *berghei* CSP-specific monoclonal 3D11, a neutralising antibody, and wild-type *P*. *berghei* challenge, data previously published in [8] but not analysed using the present model. Fig 5 shows estimates of *p*_*b*_ using curve-fitting to **(19)** (Fig 5A) and linear regression to **(14)** (Fig 5B).

The two methods give precise estimates of *p*_*b*_ and in good agreement with one another (p = 0.16 by t-test, Fig 5C). Since curve-fitting to **(19)** does not rely on a value for *g* it is taken to be more reliable and hence the estimate *p*_*b*_ = 0.022 ± 0.004 is used for simulations in the sections below.

#### Estimating pc

As with *p*_*b*_ two independent methods can be used to estimate *p*_*c*_: if only CTL are present in the system, *b* = 0 and so curve-fitting can be performed against this equation derived from **(3)**:

$$p_{S}={\left(1-\left(k*{\left(1-p_{C}\right)}^{C}\right)\right)}^{Z}$$ (21)

*p*_*c*_ can also be derived from the linear Eq **(15)** with a value for *g*. The only way to ensure CTL are the sole mediators of efficacy is by adoptive transfer of CTL; but even this method would give an inaccurate value due to the high rate of cell death associated with this method. Vaccination with viral-vectored TRAP is usually thought to be mediated chiefly or exclusively by TRAP-specific CTL. On this assumption, **(15)** can be used with time to 1% parasitaemia from TRAP vaccination experiments. But see [49–51] for evidence that antibodies against TRAP can also mediate protection.

**Fig 6:**
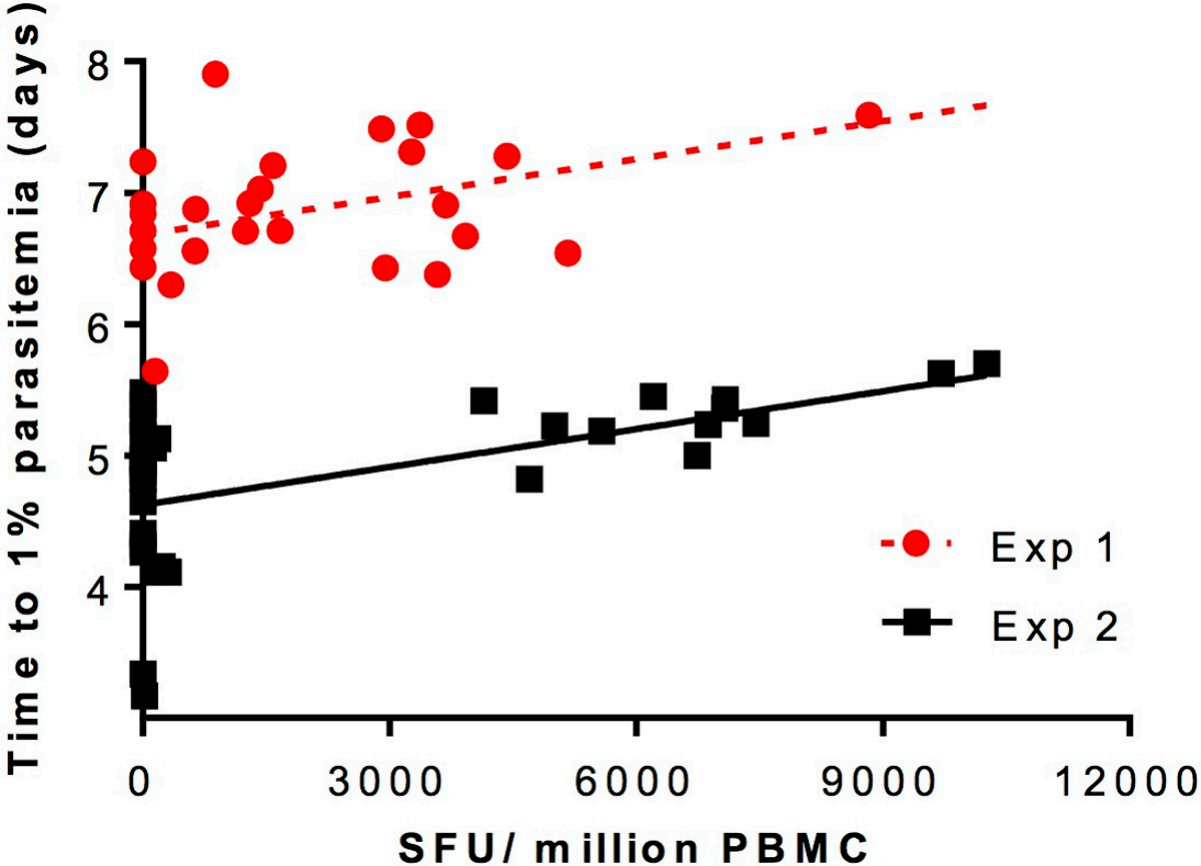
Estimating probability of protection parameter *p*_*c*_ by linear regression of SFU/million PBMC against time to 1% parasitaemia in two PvTRAP vaccination experiments. Two sets of female BALB/c mice (n = 26, 34) were vaccinated with *P*. *vivax* TRAP delivered using viral vectors (adenovirus prime and MVA boost, 8 weeks apart). ELISpots were performed using PBMCs obtained from mice 2 weeks following boost immunization. Mice were challenged one week post-boost with either 5000 (experiment 1, red circles) or 2000 (experiment 2, black squares) PvCSP/PvTRAP transgenic *P*. berghei sporozoites. Time to reach 1% blood-stage parasitaemia was obtained by linear regression using daily thin blood smears. Linear regression using Prism and analysis of the slopes returns a pooled slope value of 9.62E-05 ± 2.17E-05, with the probability of the slopes being different of p = 0.63. From the equation $T_{V}=-\frac{\log\left(1-p_{c}\right)}{\log\;g}*C+T_{N}$ where *T*_*V*_ = time-to-1% in vaccinated mice, *g* = blood stage growth rate, *C* = SFU/million PBMC, *T*_*N*_ = time-to-1% in naïve mice, and *p*_*C*_ = probability of protection by a unit of *C*, and using a value of *g* = 3.5 ± 0.1, a value of *p*_*C*_ = 1.21E-04 ± 2.72E-05 is obtained. Values shown ± SEM.

In two separate experiments, mice were vaccinated using viral-vectored vivax TRAP and challenged using the double transgenic Pv210/PvTRAP parasite (Fig 6). The two experiments give identical slopes by analysis in Prism (p = 0.63 that there is a difference in the slopes) and from the pooled slope a value of *p*_*c*_ = 1.21E-04 ± 2.72E-05 is obtained, assuming a value of *g* = 3.5.

### Preliminary validation of the model using a published study

In this section it is shown that the model presented here is consistent with the outcome of a previously published experiment from an independent research group. In that paper [52] two neutralising antimalarial antibodies were combined, one specific for the central repeat region of *P*. *falciparum* CSP, and the other for a CSP N-terminal domain-specific epitope. Table 3 shows the effect of combining these two monoclonal antibodies on 18S rRNA copies of *P*. *falciparum* CSP-replacement *P*. *berghei* in mice following challenge. To apply the model to this data further adaptation is required as shown below.

**Table 3:** Actual and predicted effects of combining anti-sporozoite mAb5D5 and mAb2A10 on liver burden of malaria.

mAb	rRNA copies	μg mAb	pb
**naïve**	5.43E+05	-	-
**mAb5D5**	1.14E+04	25	0.1432
**mAb2A10**	5.71E+04	100	0.0223
both mAbs	**rRNA copies**	**error**	
**predicted**	1.20E+03	3.52E+02	
**actual**	3.08E+03	9.83E+02	

It is assumed that the number of rRNA copies of *P*. *berghei* is linearly proportional to the number of infected hepatocytes. In that case, Eq **(11)** can be adapted thus:

$$\frac{L_{V}}{L_{N}}=\frac{rRNA_{V}}{rRNA_{N}}={\left(1-p_{b1}\right)}^{b1}*{\left(1-p_{b2}\right)}^{b2}$$ (22)

Comparing the rRNA values in mice treated with one monoclonal (rRNA_V_) to that of naive mice (rRNA_N_) provides estimates of *p*_*b*_ for each individual monoclonal, shown Table 3. A prediction of their combination can then be obtained using the same equation. The actual and predicted values of rRNA copy number are in close agreement, giving validation to the model.

### Simulations of vaccine combinations and implications for optimal vaccination strategies

**Fig 7:**
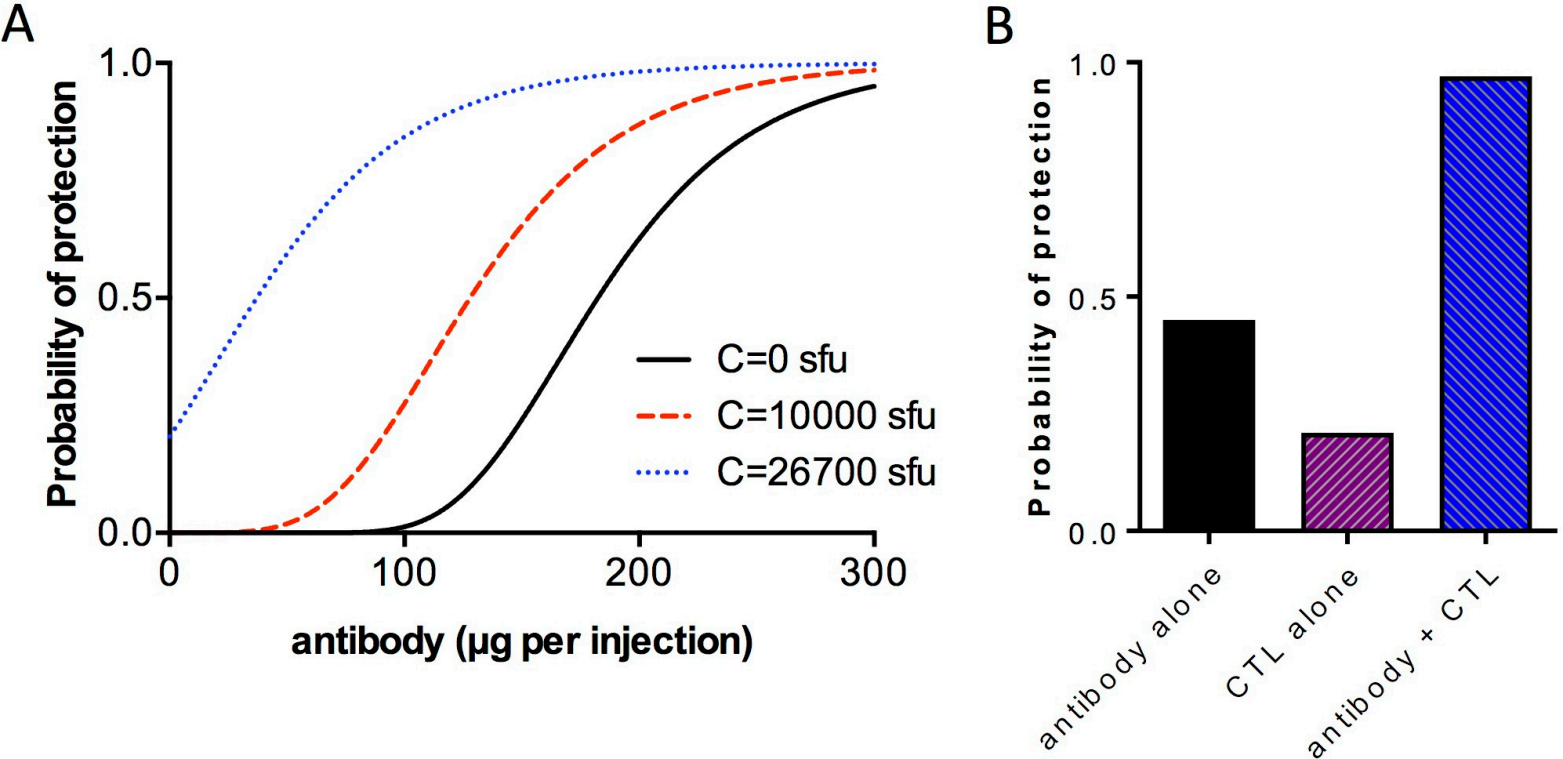
Simulation showing effects of adding a CTL-mediated vaccine to an antibody-mediated vaccine. Simulations were performed using the equation $p_{S}={\left(1-\left(k*{\left(1-p_{b}\right)}^{b}*{\left(1-p_{C}\right)}^{C}\right)\right)}^{Z}$ where *p*_*S*_ is the probability of sterile protection, *k* the natural infectivity of sporozoites, *p*_*b*_ and *p*_*C*_ the probability of protection parameters with antibodies *b* and cytotoxic T-cells *C* respectively. Using experimentally-derived values of *p*_*b*_ = 0.022 and *p*_*C*_ = 1.21E-04, (A) shows a simulation of the effect of increasing antibody concentration with various fixed concentrations of cytotoxic T-cells. (B) shows that combining an antibody-inducing vaccine of 45% efficacy with a CTL-inducing vaccine of 21% efficacy is predicted to give a protective efficacy of 97%.

Using the values of *g*, *p*_*b*_ and *p*_*c*_ obtained in above, we can simulate combinations of the two leading vaccine candidates, CSP and TRAP. If CSP efficacy were chiefly mediated by antibodies, and TRAP efficacy by CTL, combining a TRAP vaccine to a CSP vaccine would be expected to enhance protective efficacy as shown in Fig 7. Fig 7B shows that the combination of TRAP and CSP should have a more than additive effect, raising protective efficacy from 21% and 45% for TRAP and CSP to over 97%. An additive effect in this case would predict protection of 1 –(1–0.21)*(1–0.45) = 56.6%.

It is also a consequence of the model that with increasing numbers of antibody or CTL of unique epitope specificity the quantity of each antibody or CTL type required to achieve a given level of protection, for instance 80%, is reduced. If the value *p*_*b*_ of each antibody of unique epitope specificity is the same, then the sum of antibody required to achieve a certain level of protection is a constant. Since achieving moderate levels of antibody against each of a wide range of epitope targets is likely to be easier than achieving high levels of antibody against just a single target, the former is an attractive vaccine strategy.

**Fig 8:**
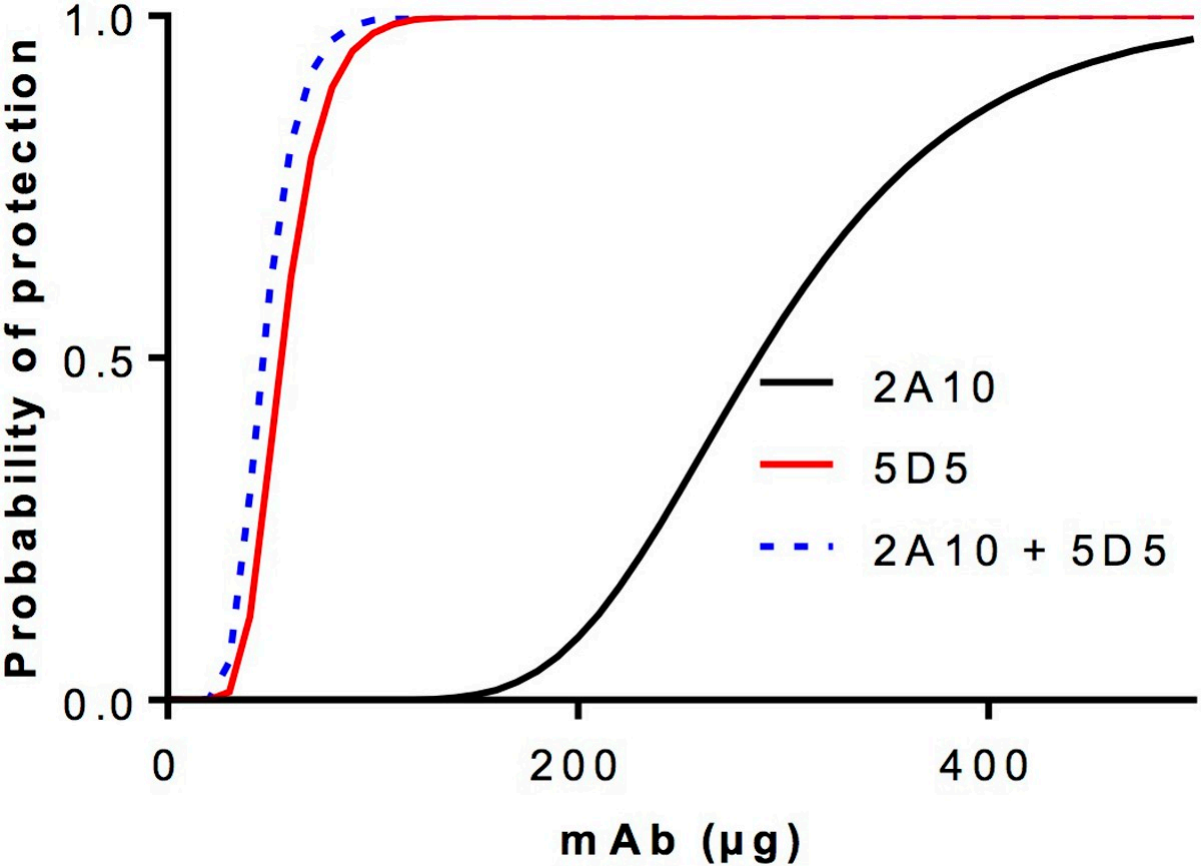
Simulation showing that a disparity in the potency of two monoclonal antibodies diminishes returns from their combination. The equation $p_{S}={\left(1-\left(k*{\left(1-p_{b1}\right)}^{b1}*{\left(1-p_{b2}\right)}^{b2}\right)\right)}^{Z}$ is used to simulate the effects of combining two antibodies of markedly different potency (probability of protection parameters *p*_*b1*_ and *p*_*b2*_, using experimentally derived values for *P*. *falciparum* CSP-specific monoclonal antibodies 2A10 (*b*_*1*_*; p*_*b1*_ = 0.022) and 5D5 (*b*_*2*_*; p*_*b2*_ = 0.143), where *p*_*S*_ is the probability of sterile protection, *k* the natural infectivity of sporozoites, and *Z* the number of sporozoites, using *Z* = 1000 and *k* = 0.04.

The previous section suggested that the *p*_*b*_ values of neutralising monoclonal antibodies can be almost an order of magnitude different from one another (see Table 3). Fig 8 demonstrates that in a conventional challenge experiment adding mAb2A10 to mAb5D5 would have virtually no measurable impact, with the CSP N-terminal specific mAb5D5 contributing almost all the overall protective efficacy.

This illustrates another important implication of the mathematical model: vaccine combination strategies will only noticeably result in enhancement of protection if the *p*_*b*_ values of the elicited antibodies are comparable. If one antibody type has a much higher *p*_*b*_ than another, then little or no enhancement of protective efficacy will be achieved by combining them. Ultimately the question of which vaccines to combine will be determined by considerations of cost-effectiveness.

### Implications for basic biology of *P*. *berghei*

The equations derived in this paper can be used to infer bounds on unknown parameters, namely *R* (the number of iRBC deriving from each infected hepatocyte), and *T*_*max*_ (the maximum time-to-1%, which occurs when only one hepatocyte is successfully infected). In addition *k* (the natural infectivity of sporozoites) can be estimated for the typical experiment, with an average time-to-1% in naïve mice of 6.7 d (*T*_*N*_ = 6.7 d) in experiments where 1000 sporozoites are inoculated (*Z* = 1000). Because these are average values over many experiments, it is assumed any experimental errors in these values are negligible, and that there is no systematic error. Another parameter, the time it takes parasites to develop in the liver before seeding the blood stage, *t*_*1*_, is taken from existing literature to be 2.1 d [53]. The growth rate *g* is taken to vary between 3.4 and 3.6 based on data presented above. *B*, the number of red blood cells in a 22 g BALB/c mouse, is taken from the literature to be 1.23E+10 [54].

On this basis, a minimum value for *T*_*max*_ can be obtained from the equation

Tmax=t1+log(B(100*R))logg (23)

derived from Eq **(9)** using *L*_*N*_ = 1 when *T*_*N*_ = *T*_*max*_. Using the bounds of *g* ≤ 3.6 and *R* ≤ 1000 [24] places a minimum bound of *T*_*max*_ ≥ 11.2 d.

A maximum bound on *T*_*max*_ is placed by *k*, the infectivity. The probability that a given sporozoite infects cannot be greater than 1. Using the equation

Tmax=TN+log(k*Z)logg (24)

derived from Eq **(19)**, using *k* ≥ 1 and the lower bound of *g* ≥ 3.4 gives a upper bound of *T*_*max*_ ≤ 12.3 d.

*R* is also bounded by *k* ≤ 1. From the equation

$$R=\frac{B}{\left(100*k*Z*\left(g^{Tn-t1}\right)\right)}$$ (25)

derived from Eq **(17)**, and using the upper bound of *g* = 3.6, a lower bound of *R* ≥ 340 is obtained.

Finally, a minimum infectivity *k* for the typical experiment is obtained using the upper bound *R* ≤ 1000, using the equation

$$k=\frac{B}{\left(100*R*Z*\left(g^{Tn-t1}\right)\right)}$$ (26)

likewise derived from **(17)**. This gives a lower bound on infectivity *k* ≥ 34%.

**Fig 9:**
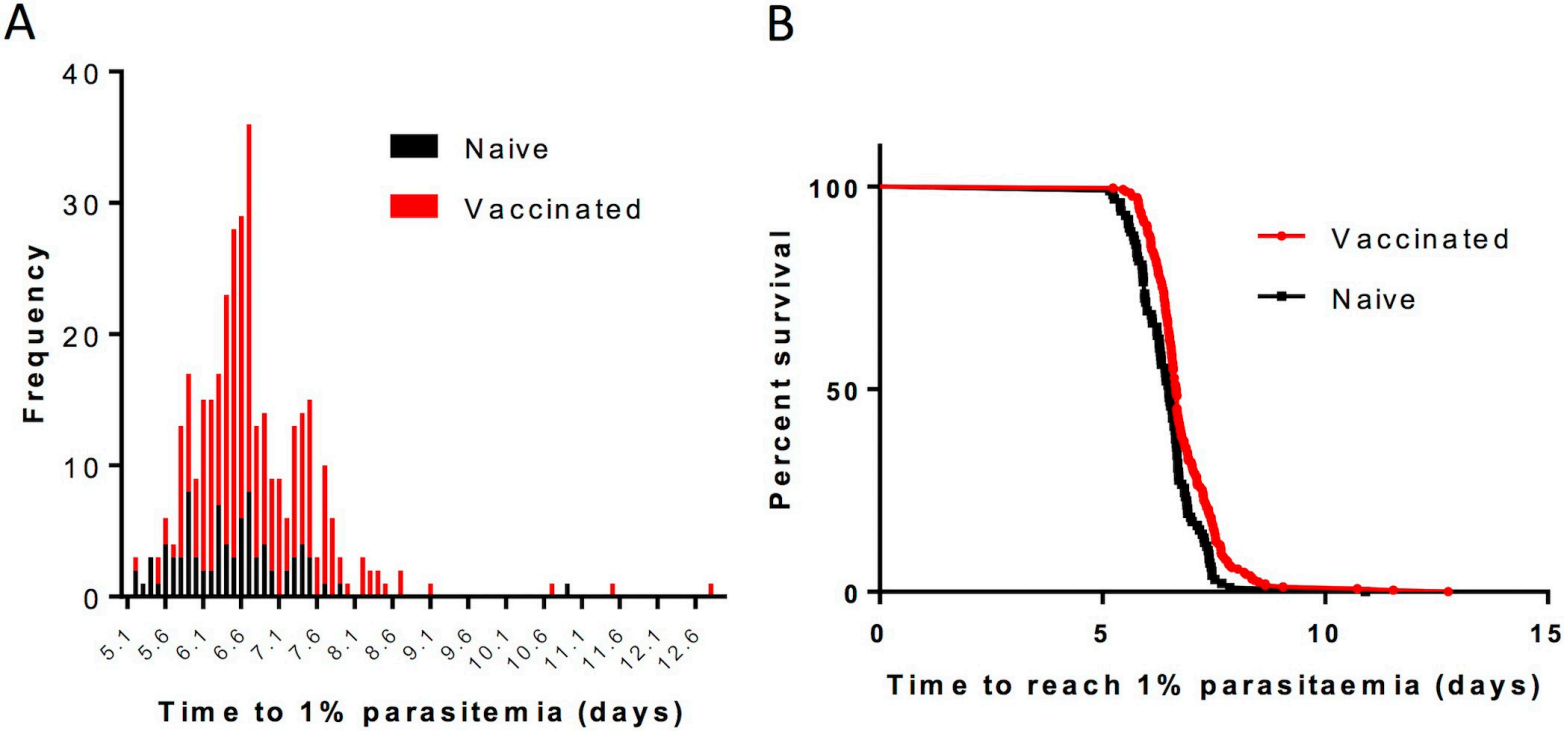
Time-to-1% values in naïve and vaccinated mice (BALB/c, 2207 parasite). Daily blood smears of naïve (n = 84, blue bars) and vaccinated (n = 269, red bars) mice challenged with varying quantities of sporozoites were used to calculate time to reach 1% blood-stage parasitaemia by linear regression analysis. (A)shows the frequency of mice reaching 1% blood stage parasitaemia at each time period (0.1 d intervals) between 5 days and 13 days post-infection. (B) represents the same data as a Kaplan Meier plot.

It is difficult to experimentally confirm *in vivo* values for *R* and *k*. On the other hand, data is available from the challenge experiments performed by the authors against which to compare the predicted value of *T*_*max*_. Fig 9 is a histogram showing the frequency of values of time-to-1% in naïve and vaccinated mice. Two vaccinated mice reach 1% parasitaemia at *T* = 11.5 and 12.8 d; and one naïve mouse at *T* = 10.9 d. Given experimental error and some natural variation in *R*, this data is at least consistent with the value for *T*_*max*_ predicted by the above bounding estimates.

Table 4. summarises the bounds placed on all variables described in this section.

**Table 4:** Inferred or actual mean, lower and upper bounds for biological parameters of *P*. *berghei* infection in BALB/c mice.

Variable	lower	mean	upper
*t*_*1*_		2.1 d	
*T*_*N*_		6.7 d	
*Z*		1000 spz	
*B*		1.23E+10 RBC	
*g*	3.4	3.5	3.6
*T*_*max*_	11.2		12.3
*R*	340		1000
*k*	0.34		1

## Discussion

In this paper, a mathematical model of vaccine combination was derived from simple probabilistic assumptions. It is designed to be of particular use in analysing and interpreting murine pre-erythrocytic malaria vaccine challenge experiments.

The model was found to fit pre-clinical data well enough to allow estimates of the fundamental parameters *p*_*b*_ and *p*_*c*_. This allowed the prediction that a CSP-like vaccine conferring 45% efficacy and a TRAP-like vaccine conferring 21% efficacy would, assuming no antigenic interference, confer a combined protective efficacy of 97%. This is in very close agreement with another CHMI model [14] which, using the same baseline vaccine parameters, predicted the combination to give an efficacy of 97.5%. In contrast, a Hill-function based model [11] is predicted to give an enhancement from 50% for one antibody-inducing vaccine to only 75% with the addition of an equally efficacious vaccine, with a similar result in [12]. The discrepancy between models is likely due to the inclusion by Saul *et al*. and White *et al*. of a component in the models accounting for the distribution of antibody responses to vaccination in a population, which probably makes these models more reliable, and hence represents a limitation in the model herein presented. Further work could expand the model to capture such variation in antibody levels across a population.

All models, however, are in agreement that combining pre-erythrocytic malaria vaccines would markedly enhance protective efficacy. Vaccine combination therefore represents an attractive strategy for improving on the low protective efficacy currently seen with the leading malaria vaccine candidate RTS,S/AS01 [55,56]. However, Saul [10] cautions that combining vaccines will only result in enhanced efficacy if they individually have comparable efficacy, a finding repeated here (Fig 8).

Antigenic interference, a phenomenon whereby the presence of an additional antigen in a vaccine diminishes the immune response normally generated against another antigen, represents the biggest obstacle to exploiting the benefits of including multiple antigens. The model presented in this paper will still correctly describe the protective efficacy of a combination vaccine based on the actual immune responses generated by each subunit vaccine in the combination formulation, but if antigenic interference has occurred the protective efficacy will fall short of the protection that could have been obtained without antigenic interference. There is still little understanding of the conditions under which antigenic interference occurs; for instance, mixing and co-injecting the vaccines into one site sometimes does [57] and sometimes doesn’t [58] result in antigenic interference, and the same goes for delivery of the component vaccines into separate sites (antigenic interference in some cases, [59], but not in others [60]). If we are to fully exploit the potential of vaccine combination predicted by the model here presented, a thorough study of the conditions under which antigenic interference occurs should be carried out. A separate motivation for including a breadth of antigens derives from variability of immune response in individuals, for instance due to wide variability in HLA haplotype between individuals, which argues for inclusion of multiple antigens in order to confer broad population-level immunity. Here too a greater understanding of antigenic interference could prove extremely useful.

A formal comparison between the model presented in this paper and those presented by White *et al*. [12,13] and Saul *et al*. [10,11] (summarised in the Introduction) reveals that the model in this paper is not mathematically equivalent to the Hill function- or exponential-based models, and hence is novel. To recapitulate, the two models are:

rij=11+(xijβj)α

and

$$r_{ij}=e\frac{-x_{ij}\log(2)}{\beta_{j}}$$

where *x*_*ij*_ is the antibody titre to a specific antigen in a given individual, *r*_*ij*_ is the probability that a sporozoite survives the immune response *x*_*ij*_, *β*_*j*_ is the antibody titre which reduces the probability of sporozoite infection by half, and α is the shape parameter of the Hill function. *r*_*ij*_ is equivalent to (1-*p*_*b*_)^*b*^ in terms of this paper’s model, where *b* is the antibody titre and so *b = x*_*ij*_. When *b = β*_*j*_, *r*_*ij*_ = ½ = (1-*p*_*b*_)^*Bj*^. Hence (1-*p*_*b*_) = (½)^1/*Bj*^ and so on the basis of my model, in the terms used by White *et al*.,

$$r_{ij}={\left(\frac{1}{2}\right)}^{x_{ij}/\beta_{j}}$$

Further work is needed to show whether this probabilistically-derived alternative to the Hill-function and exponential dose-response relationships better fits available data.

The model is derived from explicit propositions which can in principle be altered to generate other biologically-grounded models. This could enable basic assumptions about the immunology behind the model to be experimentally tested. Some experimental data has recently been published which is pertinent in this regard [61]. In that study the authors control the number of CSP-specific CD8^+^ T-cells by adoptive transfer, and the number of sporozoites used to challenge mice. Thus they are able to alter the effector to target (E:T) ratio *in vivo* at will. The authors show that mice challenged with the same E:T ratio, but greater numbers of both CD8^+^ T-cells and sporozoites, recruited more liver-resident CD8^+^ T-cells into division and were better protected against challenge. This contradicts the independence assumption that the probability *p*_*C*_ that a unit of cytotoxic T-cells has a fixed probability of preventing successful infection; instead, *p*_*C*_ may be a function of the number of sporozoites inoculated. In consequence the model may underestimate the efficacy of CD8^+^ T-cell vaccines at high challenge doses. The finding could have implications for the translation of pre-clinical malaria vaccine combination studies to human trials. Specifically, if a combination of pre-erythrocytic vaccines are found to substantially enhance protection in combination, because they are comparatively protective against a high challenge dose in mice, it is possible that the enhancement in protection from combination may not be as significant with lower challenge doses in the context of a clinical or field trial. It also remains to be seen whether this is the case and, if so, the model here presented can be modified to incorporate this finding by rejecting the independence assumption concerning *p*_*C*_, resulting in a more complex but better-fitting model.

Strategies designed to increase the number of malaria-antigen specific cytotoxic T-cells in the liver ([62–64]) also have implications for the model presented here. When successful, such strategies will increase the number of CTLs or resident memory T-cells in the liver, possibly at the expense of circulating PBMCs, depending on the mechanism being exploited; in either case, the number of circulating antigen-specific PBMCs could appear to predict lower efficacy when compared to a vaccine that is not using a ‘pull’ strategy to increase CTLs in the liver. Thus it is important to consider the mode of administration of a vaccine when using the present model to make comparisons between the protective efficacy of vaccines delivering the same antigens.

The value for the growth rate, *g*, obtained in this study should be reliable within the context of the model. However, it will not necessarily agree with other calculated values, which are typically higher than our estimate of 3.5 [65–69]. There could be various reasons for this. Blood stage growth rate appears to decrease with increasing parasite density [65]; sequestration has also been proposed to affect calculations of values for the blood stage growth rate [69]. In *P*. *falciparum* it has been shown that the parasite multiplication rate *in vitro* varies between strains of parasite [70], so it is possible that it may vary between clones of *P*. *berghei* also, although in the present study no difference between three transgenic clones and wild-type *P*. *berghei* was found (Fig 1). Values for the number of merozoites per iRBC, for instance a range of 16–18 in reticulocytes (from LUMC), suggest that if this value of *g* is correct, *P*. *berghei* merozoites have an infectivity of about 20% in mice.

Inaccuracy in the value of *g* would not affect our main conclusions, but could affect estimates of the values of other biological parameters derived using the model. Sequestration, the most likely reason for a discrepancy between my value and other published values of *g*, would cause the values for *R* and *T*_*max*_ to be underestimated. Another potential limitation of the model is the simplifying assumption that merozoite release from infected hepatocytes occurs approximately simultaneously. Since blood-stage growth, particularly at low levels of parasitaemia, is exponential, early-release hepatocytes will tend to dominate the blood-growth stage, and hence the assumption of simultaneity of release is appropriate. However, the assumption of simultaneity, if very inaccurate, would again cause our inferred value of R to be an underestimate. Nevertheless, there is reason to believe that the values estimated for *R*, *T*_*max*_ and *k* (the infectivity of sporozoites in naïve mice) are of approximately the correct magnitude. In all the experiments performed by the first author of this paper, the maximum time to 1% parasitaemia values have been quite close to that of the predicted value for *T*_*max*_, 11–12 days (Fig 9). Furthermore, a high value for *g* is incompatible with high infectivity, and a low infectivity is incompatible with, for instance, a dose of 150 sporozoites per mouse giving 100% infection in 18 mice, as was the case in one experiment performed by the authors; so the low value of *g* and the high value of *k* here presented are at least consistent with our own experimental results. Published data on sporozoite infectivity to mouse livers *in vivo* has traditionally put the value quite low: 0.02% for BALB/c and 8.2% for C57BL/6 mice [71], and 3.8% for A/J mice [72]. These estimates were later criticised for making inaccurate assumptions about the size and shape of infected hepatocytes, and a revised infectivity of 1%– 1.6% for C57BL/6 mice was calculated [73]. All these estimates for infectivity are much lower, however, than our own minimum bound on infectivity of 34% in BALB/c mice. Two factors may help explain this. The first is that previous estimates have assumed a uniform distribution of infected hepatocytes throughout the liver, whereas sporozoites tend to infect hepatocytes around portal venules [74,75]. Secondly, improved techniques for the dissection of mosquito salivary glands to obtain sporozoites, in particular the use of Schneider’s media lacking activating bicarbonates which increase sporozoite death [76], may have resulted in greater infectivity of sporozoites than obtained historically. Improvements in sporozoite purification are also likely improving infectivity compared to historical techniques [77,78]. Thus the infectivity of 34% we derive may be plausible.

In summary, a novel mathematical model based on simple probabilistic assumptions has been developed in this paper. It expresses either probability of sterile protection, or time to reach 1% blood-stage parasitaemia, as a function of quantities of antibody or cytotoxic T cells, and is validated by application to previously published data. The model represents a theoretical advance on previous work which does not derive a dose-response relationship from basic biological assumptions. Moreover, it demonstrates that malaria vaccine combinations should, if antigenic interference can be avoided, markedly enhance efficacy compared to either component alone, and hence should be pursued as a strategy for developing the next generation of malaria vaccines.

## Materials and methods

### Thin blood smears and calculation of time to reach 1% blood stage parasitaemia

Thin blood smears, taken daily, were prepared on glass slides from a drop of blood from the tail tip of challenged mice. Slides were fixed in methanol then stained in 5% Giemsa (Sigma) for 30 min and washed in water. 1000 red blood cells were counted for three to five consecutive days until the mouse reached 1% blood stage parasitaemia. Time to reach 1% blood stage parasitaemia was calculated by linear regression of log_10_ percentage parasitaemia against time post-challenge, as described in [34]. Mice without parasites by day 15 were considered to have been conferred sterile protection against challenge. The data set of time-to-reach-1%-parasitaemia upon which a value for blood stage growth rate is inferred for various strains of mice, as well as for vaccinated and unvaccinated mice (Fig 1), derives from murine malaria vaccination/challenge experiments conducted by EA during his D.Phil thesis.

### Vaccinations

The mice described as ‘vaccinated’ in Fig 1 were administered CSP- or TRAP-based vaccines, either as protein-in-adjuvant, Hepatitis C surface antigen-based virus-like particles, or adenovirus- or MVA-based viral vectors, as described in more detail in Atcheson *et al* [9].

### Dissection of mosquito salivary glands and challenge of mice with sporozoites

21 days after feeding on *P*. *berghei*-infected mice, mosquitoes were sedated at 4°C for dissection. Salivary glands were dissected from mosquitoes under a microscope and removed by pipette into a glass tissue homogeniser containing 100 μL Schneider’s insect media with 10% FBS. Sporozoites were liberated from salivary glands by gently homogenising three times and counted using a haemocytometer. Sporozoite concentration was typically adjusted to 10^4^ sporozoites/mL for intravenous injection into the tail vein of mice of 100 μL (1000 sporozoites per dose, by insulin syringe) unless otherwise stated. Mosquitoes were obtained from the Jenner Institute insectary, University of Oxford.

### Production of transgenic P. berghei

Pv-CSP/PvTRAP (“2207”), PfCSP and PvCSP-247 transgenic *P*. *berghei* parasites were produced as described in [9,79,80]

### Serial dilutions of infected red blood cells and sporozoites to determine blood-stage growth rate

Serial dilutions of infected red blood cells (iRBC) and sporozoites were performed for curve fitting to equations described in this paper.

iRBC were obtained from unvaccinated donor BALB/c mice with > 1% blood-stage parasitaemia by cardiac bleed with a 25G needle and syringe containing 2–3 μl heparin. A thin blood smear was taken from this mouse for back-calculation of undiluted percent parasitaemia. Blood was immediately diluted 1:2 in a series of nine in PBS (Sigma) and 100 μl of each dilution injected intravenously into the tail vein of a BALB/c mouse. Calculation of the number of iRBC injected into each mouse was derived from donor mouse parasitaemia and on the assumption of 1.21x10^10^ RBC per 20 g BALB/c mouse (607).

Sporozoites from mosquito dissection were counted and serial diluted in Schneider’s insect media to give, in one set of experiments, a series of 10000, 6667, 3333, 1667, 833 and 417 sporozoites per dose; and in another series of experiments, 16000, 8000, 4000, 1000, and 500 per dose.

### Injection of 3D11 monoclonal antibody

3D11 monoclonal antibody was obtained and injected into mice as described in [8]

### Mouse PBMC IFNγ ELISpot

IFNγ ELISpots were performed as previously described (605). The BALB/c PvTRAP immunodominant peptide required for stimulation of PvTRAP-specific CD8+ T-cells was identified in (497) and is ITKVIPMLNGLINSLSLSRD.

### Mouse strains used

6 week-old female BALB/c (H-2d) mice and C57BL/6 (H-2b) mice were used for vaccination experiments, with age-matched controls. TO outbred mice and BALB/c mice were used for parasite maintenance and mosquito feeds. All mice from Harlan/Envigo.

### Ethics statement

All animals and procedures were used in accordance with the terms of the United Kingdom Home Office Animals Act Project License. Mice were euthanized by cervical dislocation following experimental use. The procedures were approved by the University of Oxford Animal Care and Ethical Review Committee (PPL 30/2889 and P9804B4F1).

### Statistical analysis

GraphPad Prism (MacOS v6) and Microsoft Excel were used for all statistical analyses performed. Student’s t-test and ANOVA with Bonferroni’s multiple comparisons test were used on parametric data comparing two or more groups respectively. The non-linear curve-fitting function in GraphPad Prism was used to fit specified equations to data.

## Supporting information

S1 Table The raw data for all figures and simulations is presented in tabular form in this set of supplementary tables. (XLSX)
